# *Clostridioides difficile* Single Cell Swimming Strategy: A Novel Motility Pattern Regulated by Viscoelastic Properties of the Environment

**DOI:** 10.3389/fmicb.2021.715220

**Published:** 2021-07-21

**Authors:** Julian Schwanbeck, Ines Oehmig, Uwe Groß, Andreas E. Zautner, Wolfgang Bohne

**Affiliations:** Institute for Medical Microbiology and Virology, University Medical Center Göttingen, Göttingen, Germany

**Keywords:** *Clostridioides difficile*, motility, viscoelastic medium, video microscopy, motility tracking, bacterial swimming strategy

## Abstract

Flagellar motility is important for the pathogenesis of many intestinal pathogens, allowing bacteria to move to their preferred ecological niche. *Clostridioides difficile* is currently the major cause for bacterial health care-associated intestinal infections in the western world. Most clinical strains produce peritrichous flagella and are motile in soft-agar. However, little knowledge exists on the *C. difficile* swimming behaviour and its regulation at the level of individual cells. We report here on the swimming strategy of *C. difficile* at the single cell level and its dependency on environmental parameters. A comprehensive analysis of motility parameters from several thousand bacteria was achieved with the aid of a recently developed bacterial tracking programme. *C. difficile* motility was found to be strongly dependent on the matrix elasticity of the medium. Long run phases of all four motile *C. difficile* clades were only observed in the presence of high molecular weight molecules such as polyvinylpyrrolidone (PVP) and mucin, which suggests an adaptation of the motility apparatus to the mucin-rich intestinal environment. Increasing mucin or PVP concentrations lead to longer and straighter runs with increased travelled distance per run and fewer turnarounds that result in a higher net displacement of the bacteria. The observed *C. difficile* swimming pattern under these conditions is characterised by bidirectional, alternating back and forth run phases, interrupted by a short stop without an apparent reorientation or tumbling phase. This motility type was not described before for peritrichous bacteria and is more similar to some previously described polar monotrichous bacteria.

## Introduction

*Clostridioides* (formerly *Clostridium*) *difficile* is a spore forming, obligate anaerobic pathogen that causes CDI (*C. difficile* infection), which predominantly manifests as hospital-associated diarrhoea and pseudomembranous colitis (Leffler and Lamont, 2015; Lessa et al., 2015). Clinical symptoms are associated with toxin expression, in particular toxin A and B, which undergo a complex regulation pattern (Just et al., 1995; Kuehne et al., 2010; Chandrasekaran and Lacy, 2017). An intact gut microbiome is believed to protect from *C. difficile* infection. Dysbiosis however, for example after antibiotic treatment, favours *C. difficile* spore germination and subsequent colonisation (Buffie and Pamer, 2013; Theriot and Young, 2015; Gómez et al., 2017).

Flagellar motility and chemotaxis are important for successful colonisation and virulence of many gastrointestinal pathogens, for example *Campylobacter jejuni*, *Salmonella enterica* Serovar Typhimurium, *Helicobacter pylori*, and *Vibrio chlolerae* (Boin et al., 2004; Stecher et al., 2004; Lertsethtakarn et al., 2011; Korolik, 2019). Most *C. difficile* strains produce peritrichous flagella, which can mediate swimming motility in soft-agar based assays (Twine et al., 2009; Baban et al., 2013; Courson et al., 2019). The contribution of flagellar motility for the pathogenesis in mice was studied with the aid of *C. difficile* flagellar mutants, which were found to be reduced in their colonisation efficiency (Baban et al., 2013; Batah et al., 2017). It is also known that the *C. difficile* genome contains a region which encodes for a complete set of chemotaxis genes (Dannheim et al., 2017). Chemotaxis allows bacteria to swim up or down a chemical gradient and thus to find optimal growth conditions (Matilla and Krell, 2018). Regulation of motility by the chemotaxis system is well investigated for a variety of bacterial species. Chemical gradients are sensed by chemoreceptors, which transfer the signal via the adapter protein CheW and the histidine kinase CheA to the transducer CheY, which becomes phosphorylated (Porter et al., 2011). The phosphorylated CheY finally interacts with the flagellar motor and leads to a modulation of motility characteristics.

However, in contrast to other gut pathogens, little knowledge exists on the motility of *C. difficile* at the single cell level. A careful analysis of the *C. difficile* swimming pattern and its dependency on environmental parameters would contribute to a better understanding of *C. difficile* motility for pathogenesis and particularly for the attachment and dissemination phases, in which flagella have previously been found to play a role (Tasteyre et al., 2001; Batah et al., 2017).

Gut pathogens like *C. difficile*, as well as commensalists, have to deal with the mucosal layer in the lower intestine. The colon mucus, made up mostly of Muc2, consists of a stratified inner, attached layer which is expressed by the surface goblet cells and serves as a protective barrier for the intestines (Lai et al., 2009; Johansson et al., 2011; McGuckin et al., 2011). The pore sizes of this layer allow for small molecule diffusion, but acts as a barrier to structures in the micrometre range, partially due to steric hindrance (Lai et al., 2009). The mucus layer is partial degraded by commensalists and possesses then an increased pore size, allowing a better colonisation by bacteria (Lai et al., 2009; Johansson et al., 2011; McGuckin et al., 2011). As the layer is still cross-linked, it presumably still poses as a steric hindrance, influencing the rheological profile of the environment.

We report in this study on the swimming behaviour of *C. difficile* and its strong dependency on the viscoelastic properties of the medium across all motile clades. A comprehensive quantitative analysis of *C. difficile* motility parameters on the single cell level was obtained with the aid of the bacterial tracking programme YSMR (Schwanbeck et al., 2020). We hypothesise that this dependency on viscoelastic properties is an adaptation to the properties of the mucin rich lower intestines, which form the habitat of *C. difficile*. A large-scale quantitative analysis of the swimming behaviour leads to the conclusion that the single cell motility displayed by *C. difficile* forms a novel pattern for peritrichous bacteria, which we describe here in detail.

## Materials and Methods

### Used *C. difficile* Strains

*C. difficile* 630 Δ*erm* [Ribotype (RT) 012, DSM 28645, CP016318.1 (Dannheim et al., 2017)], *C. difficile* R20291 (RT 027, DSM 27147, CP029423.1), DSM 100002 (RT 084), DSM 102978 (RT not determined, CP020380.1), DSM 28670 [RT SLO 237, CP012312.1 (Riedel et al., 2017)], and DSM 100005 (RT SLO 235).

### Media and Strain Cultivation

*C. difficile* strains were grown at 37°C in BHIS (37 g/l brain heart infusion broth supplemented with 5 g/l yeast extract and 0.3 g/l cysteine) shaking at 180 rpm for liquid cultures, alternatively with 15 g/l agar for plates, or on Columbia agar with 5% sheep blood (COS, bioMérieux, Nürtingen, Germany). Cultivation was always performed under anaerobic conditions using a COY anaerobic gas chamber (COY Laboratory Products, Grass Lake, United States). The chamber was gas-flushed with 85% N_2_, 10% H_2_, and 5% CO_2_.

### Motility With Polyvinylpyrrolidone (PVP) or Mucin

For experiments with high molecular weight polymers K 90 polyvinylpyrrolidone (MW 360,000 g/mol, Carl Roth, Karlsruhe, Germany, order nr. CP15.1) and Type I-S mucin (Bovine Submaxillary glands, Merck, Darmstadt, Germany, order nr. M3895-100MG) was used. From a PVP or Mucin stock solution (see Supplementary Text 2), the desired end-concentration of the additive as stated in the experiment was prepared in Dulbecco’s phosphate buffered saline (PBS, Merck, Darmstadt, Germany, order nr. D5652) to a volume of 90 μl. The solution was then incubated anaerobically at 37°C for at least 1 h. From a mid-exponential culture (OD_600_ 0.4–0.6) 1 ml was centrifuged at 1,500 × g for 15 min using slow acceleration and deceleration ramps during centrifugation. The supernatant was removed, and the pellet resuspended to an OD_600_ of 6 in anaerobised BHIS. From the bacterial suspension, 10 μl were added to the PVP/Mucin solution for a final volume of 100 μl and OD_600_ of 0.6.

### Video Microscopy

Inside the anaerobic chamber, 4 μl of the culture were placed on an objective slide, covered with a cover slip and immediately sealed with nail polish. Except when stated otherwise, slides were recorded 15 min after sealing on a microscope at room temperature with a 10x phase contrast objective (Nikon Eclipse TE2000-S, Nikon PlanFluor 10x). Videos were recorded for 3 min at 30 fps using an Aptina CMOS Sensor 18MP 1/2.3” Colour.

For 64x phase contrast objective movies, a Leica DMR microscope with a PL APO 506082 64x objective was used. Movies were taken with a Nikon D7100 camera at 30 fps.

### Single Cell Tracking With YSMR

For quantitative data of the *C. difficile* swimming parameters, we recorded video files and always analysed 10 s of motility parameters per detected bacteria with the aid of the recently developed bacterial tracking programme YSMR (Schwanbeck et al., 2020). We used a low magnification objective (10x) for video recording in order to monitor the swimming behaviour of a large number of bacteria (100–5,000) per video. All videos were analysed using YSMR v0.1.1 using the settings as set in the “Supplementary 1_tracking.txt” file.

### Turnaround Calculation

Turnarounds were calculated using YSMR v0.1.1. Briefly, turnarounds were determined by calculating the difference in heading direction for two consecutive points. Heading directions were calculated by taking the arctan2 between the original point and one 0.3 s in the future. Turnarounds were calculated to be at positions with a relative angle change of at least 30° and an average minimum speed of at least 0.03 μm/s. The position with the greatest local angle difference was chosen as the turnaround.

### Displacement Calculation

The maximum Euclidean distance between any two points reached during the analysed period of the track was calculated.

### Calculations, Graphs and Video Editing

All performed calculations and graphs were created using Python 3.9.1, Seaborn 0.11.1 (Waskom, 2021), Matplotlib 3.4.1 (Hunter, 2007), SciPy 1.6.3 (Virtanen et al., 2020), Numpy 1.20.0 (Harris et al., 2020), and Pandas 1.2.1 (McKinney, 2010; Reback et al., 2021). Videos were edited using openCV-contrib-python 4.5.1.48 (Bradski, 2000).

## Results

### *C. difficile* Swimming Motility in BHIS Medium

When *C. difficile* 630 Δ*erm* swimming behaviour was analysed in 100% BHIS medium or in a 10% BHIS 90% PBS mixture, the bacteria could be classified into two groups. A fraction of up to 75% was completely non-motile, whereas the motile fraction displayed an unusual motility phenotype, which is characterised by alternating, short, back and forth run phases of 0.5–3 bacterial lengths, corresponding to ∼3–15 μm (Supplementary Movie 1). The frequency of directional changes is very high with ∼18 turnarounds/10 s and we thus refer to this behaviour as “jitter motility.” None of the bacteria displayed prolonged run phases and there is no substantial net displacement of the bacteria during movement. The motility of further five *C. difficile* strains from different clades was analysed under the same conditions. The clade 5 strain RT078, which is non-flagellated and non-motile in soft-agar assays and lacks a flagellar operon, was as expected completely immotile. All other tested strains, namely 630 Δ*erm* (clade 1), DSM 100002 (clade 1), R20291 (clade 2), DSM 102978 (clade 3), DSM 28670 (clade 4), and DSM 100005 (clade 4) displayed the jitter-motility phenotype in BHIS medium (Supplementary Movie 1). When *C. difficile* 630 Δ*erm* motility was analysed in 100% PBS instead of medium containing BHIS, the jitter-motility fraction was absent and bacteria were completely non-motile (data not shown).

### The Motility Phenotype Changes in the Presence of the High Molecular Weight Polymers PVP and Mucin

It is well investigated that the motility of bacteria depends on the physical properties of the medium, particularly on the viscosity and on the matrix elasticity (Gao et al., 2014; Martinez et al., 2014). *C. difficile* 630 Δ*erm* cells were exposed to various glycerol concentrations from 0.05 to 25% in BHIS medium in order to increase the viscosity. None of these conditions induced increased displacement and the bacteria remained in their jitter-motility stage.

As *C. difficile* displays motility in agarose (Baban et al., 2013; Courson et al., 2019), we wondered if altering the matrix elasticity of the medium has an influence on the motility phenotype. High molecular weight polymers such as polyvinylpyrrolidone (PVP), which increase the matrix elasticity, were described to enhance motility in various bacteria in a concentration dependent manner (Schneider and Doetsch, 1974; Greenberg and Canale-Parola, 1977). When *C. difficile* was resuspended in a solution containing 3.6% PVP, bacteria displayed a dramatic change in their motility phenotype with long, alternating back and forth runs (Supplementary Movies 2, 3). In this setup, motility could be observed for at least 2 h. When motility was analysed in 3.6% PVP dissolved in pure PBS without any BHIS, cells were completely immotile, suggesting that a nutrient source is required for the observed motility phenotype. We also tested the combination of 3.6% PVP and 10% glycerol in 10% BHIS. The addition of glycerol had no influence on the motility behaviour, indicating that glycerol did not inhibit the PVP-induced motility phenotype with long, alternating back and forth runs.

To exclude the possibility that the observed motility patterns are influenced by the relatively small distance between the microscope slide and the cover slide or the wall effect (Magariyama et al., 2005), we monitored the swimming behaviour also on cavity slides. The same motility pattern as with the original setup was observed for experiments with PVP (Supplementary Movie 9).

In order to investigate how common the observed motility phenotype is in *Clostridiodes*, we analyzed representative members from all four motile clades (Griffiths et al., 2010). The PVP-induced change of the motility phenotype occurred in all motile strains that were tested, including 630 Δ*erm* (clade 1, Supplementary Movies 4–7), DSM 100002 (clade 1), R20291 (clade 2), DSM 102978 (clade 3), DSM 28670 (clade 4), and DSM 100005 (clade 4) (Supplementary Movie 8).

We applied single cell tracking using the recently developed bacterial tracking programme YSMR (Schwanbeck et al., 2020) to analyse and quantify the motility characteristics of *C. difficile* (see Supplementary Videos 5–7). Per track we quantified the average speed, maximal displacement to body length ratio, arc-chord ratio, and calculated the turnarounds. The displacement/bacterial body length ratio indicates how much ground a bacterium covered in relation to its own length. The arc-chord ratio, calculated by dividing the length between start and end point by the total distance travelled, denotes the tortuosity of the bacterial movement, with a straight travel path resulting in an arc-chord ratio of 1.

**FIGURE 1:**
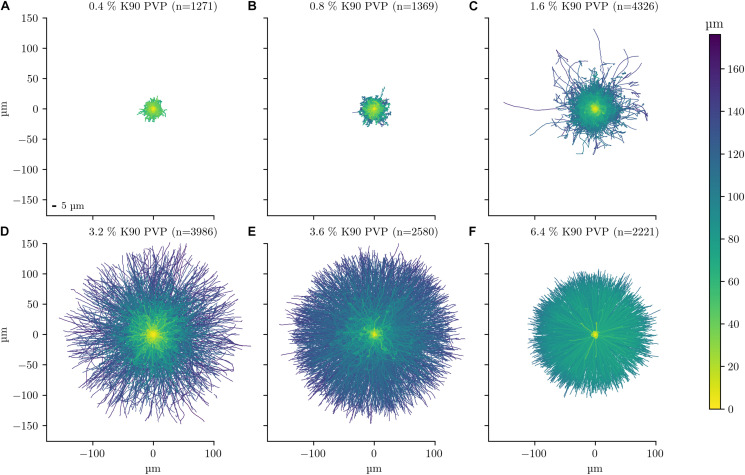
Single cell motility dependency on PVP of *C. difficile* 630 Δ*erm*. The motility of *C. difficile* cells was tracked in the presence of six increasing K90 PVP concentrations (w/v) from 0.4 to 6.4% **(A–F)**. All observation durations were limited to 10 s for better comparability. The number of displayed tracks is stated in the title of each experiment. The starting position of each track is at the origin of the plot at position 0 μm/0 μm. The scale is identical for all six plots. Track colours denote the total travelled distance, with a colour bar on the right-hand side as a legend. A 5 μm scale bar is depicted in the lower left of subfigure **(A)**. The median cell lengths of *C. difficile* was determined to be between 4.3 and 5.7 μm (Supplementary Table 1).

**FIGURE 2:**
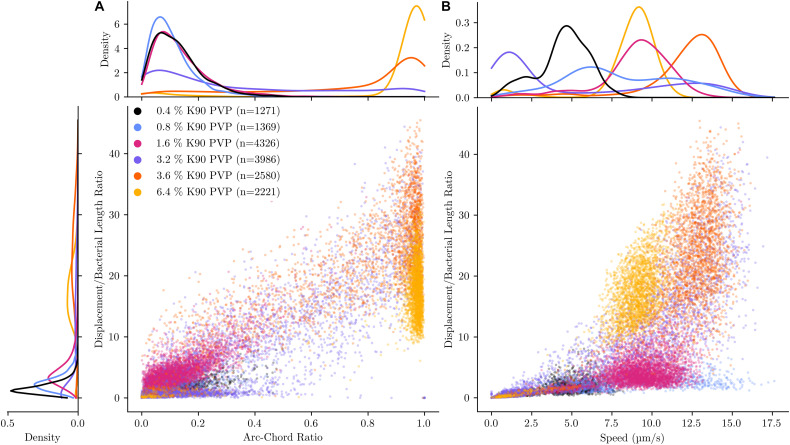
Direct comparison of motility characteristics and statistical distribution within experiments for different PVP concentrations. The motility of *C. difficile* was tracked in the presence of six increasing K90 polyvinylpyrrolidone (PVP) concentrations. The PVP concentration starts at 0.4% and increase at a doubling rate up to 6.4%, with an additional step of 3.6% PVP. All observations were limited to 10 s for better comparability. Average speed **(A)** and arc-chord ratio (**B**, denoting tortuosity of path) are plotted against the displacement/bacterial length ratio (showing the maximal distance in body lengths each bacterium has covered) for each tracked bacterium. The kernel density estimate is plotted independently for each experiment and variable, denoting the distribution for each characteristic.

To provide a graphical overview of the PVP-induced motility change, the track of each bacterium was displayed in a rose plot for an experiment with PVP concentrations in the range between 0.4 and 6.4% (Figure 1). More than 1,000 tracks were analysed for each PVP concentration. Each track is displayed with its starting position set to the origin of the coordinate system (0, 0). Additionally, each tracks maximum distance is indicated by its colour. For each track three selected characteristics are displayed in a bivariate scatterplot (Figure 2). The arc-chord ratio and average speed of each track are each plotted against the displacement/bacterial length ratio, with individual kernel density estimates for each condition. The experiment was repeated twice on different days and for each biological replicate independent rose and scatter plots were generated (Supplementary Figures 1–4).

At 0.4 and 0.8% PVP the average speed of the bacteria increases (median values 3.1–4.7 μm/s and 5.84–8.88 μm/s, respectively, Table 1) with a corresponding, albeit small increase in displacement/body length ratio (Figures 1A,B, 2). The motility phenotype at both concentrations is likely still futile, as the bacteria do not achieve notable displacement, with median displacement to length ratios below 1.5 for 0.4% and 2.5 for 0.8% PVP (Figures 1A,B and Table 1). This also is noticeable in the arc-chord ratios, which are on median between 0.08 and 0.11 (Figure 2A and Table 1). None of the bacteria revealed run phases of greater than 10 s, as can be seen in samples with higher PVP concentrations.

**TABLE 1:** Median values for select properties for different PVP concentrations.

Condition	Median displacement/Bacterial length ratio	Median distance (μm)	Median speed (μm/s)	Median arc-chord ratio
0.4% K90 PVP (*n* = 1,271, 1,300, 1,188)	1.40, 1.38, 0.80	46.69, 46.96, 30.99	4.67, 4.70, 3.10	0.11, 0.10, 0.09
0.8% K90 PVP (*n* = 1,369, 2,486, 2,251)	2.35, 2.41, 1.48	74.84, 88.77, 58.36	7.48, 8.88, 5.84	0.09, 0.09, 0.08
1.6% K90 PVP (*n* = 4,326, 2,448, 2,034)	3.77, 1.26, 2.24	92.67, 38.20, 55.70	9.27, 3.82, 5.57	0.12, 0.10, 0.13
3.2% K90 PVP (*n* = 3,986, 2,626, 2,208)	1.15, 16.57, 12.85	21.24, 109.04, 93.01	2.12, 10.90, 9.30	0.22, 0.90, 0.92
3.6% K90 PVP (*n* = 2,580, 2,416, 2,447)	21.67, 6.29, 5.60	126.28, 58.68, 56.81	12.63, 5.87, 5.68	0.90, 0.47, 0.44
6.4% K90 PVP (*n* = 2,221, 1,454, 328)	16.80, 0.16, 0.07	90.50, 7.70, 5.15	9.05, 0.77, 0.51	0.97, 0.06, 0.03

*Values correspond to Figure 1 and Supplementary Figures 1, 3 (first, second, and third given value, respectively). For a full set, including average, 25 and 75 percentiles for each property, as well as distance, displacement, and bacterial length see Supplementary Table 1.*

At 1.6% PVP the first visible shift in the motility phenotype occurs. Several individual bacteria achieve clear displacement (Figure 1C), indicating that the increase in PVP starts to form a medium in which *C. difficile* can be motile. Speed at 1.6% PVP is markedly increased in comparison to lower PVP concentrations (Figure 2B). On the population level though, bacteria still have a median displacement/bacterial length ratio between 1.3 and 3.8, and median arc-chord ratios between 0.1 and 0.13 (Table 1). The fraction of bacteria with continuous runs of at least 10 s was between 13 and 58%.

At PVP concentrations of 3.2% and higher, the majority of *C. difficile* cells display clear motility (Figures 1C–F, 2). Instead of the futile short movements, long, sustained run phases with varying curvature and intermittent turnarounds are the majority. They lead to a motility phenotype that can achieve displacement from the starting position. The motility phenotype is achieving an optimum in terms of displacement/bacterial length ratio, speed, and arc-chord ratio at around 3.2–3.6% (w/v) PVP (Figures 1D,E, 2 and Supplementary Figures 1–4). The fraction of bacteria with continuous run lengths of at least 10 s was between 67 and 86%.

At a concentration of 6.4% PVP the proportion of motile bacteria decreases in the majority of experiments (Figures 1F, 2A and Supplementary Figures 1–4). Bacteria that are still motile display very straight runs with little curvature and a median arc-chord ratio of up to 0.97 (Table 1). The fraction of bacteria showing continuous runs of at least 10 s was between 95 and 100%. The speed of motile bacteria is also markedly lower than with 3.6% PVP (Figure 2 and Supplementary Figure 2).

*C. difficile* inhabits areas with high molecular weight polymers in the larger intestine such as mucins, which cover the epithelial cell layer with a gel like structure (McGuckin et al., 2011; Semenyuk et al., 2015). We used bovine submaxillary gland mucin in concentrations ranging from 2.5 to 40 mg/ml in doubling steps (Supplementary Movie 3). The track of each bacterium was displayed in a rose plot from an experiment with mucin concentrations between 2.5 and 40 mg/ml (Supplementary Figures 7–9). At concentrations of 2.5–10 mg/ml, typical short back and forth motion was observed as in medium without PVP or at low PVP concentrations (Supplementary Figures 7–9A–C). However, at mucin concentrations of 20 and 40 mg/ml bacteria displays long, alternating back and forth run phases with few turning points and high net displacement (Supplementary Figures 7–9D,E).

### *C. difficile* Displays a Distinct Motility Phenotype

**FIGURE 3:**
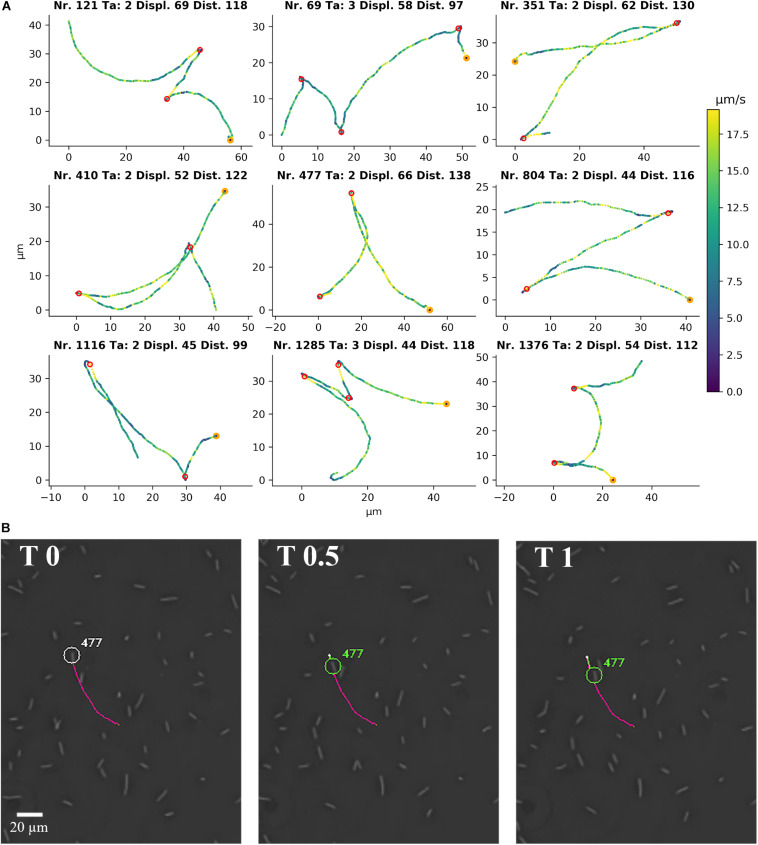
Single cell motility of *C. difficile* 630 Δ*erm* in 1.8% PVP. **(A)** Representative single cell tracks from Supplementary Movie 4 as detected by YSMR. Titles denote track number, number of turnarounds (Ta), displacement (Displ., μm), and total distance covered (Dist., μm). Colour of the positions indicates current speed in μm/s, with a colour bar on the right for reference. The start of the track is marked in orange. Red circles show the calculated positions of turnarounds. The bacteria associated with the displayed tracks are labelled in Supplementary Movie 4. The formation of tracks is shown in Supplementary Movie 4 by the extension of a coloured line behind the respective bacterium. **(B)** A picture sequence taken from Supplementary Movie 4 shows the first turnaround of track number 477, starting with the turnaround itself (T 0 s) and images at 0.5 and 1 s after the turnaround. The picture sequence shows the start of the backward movement without previous reorientation at the turnaround.

Representative tracks from a video (Supplementary Movie 4) of *C. difficile* 630 Δ*erm*, as identified by the YSMR software, are shown in Figure 3A. The swimming motility phenotype in PVP is characterised by alternating back and forth run phases, which are indistinguishable in length and duration. There is a short stop between two run phases, but there is neither an obvious tumbling nor a sudden reorientation during the stop (Figures 3A,B). Nevertheless, the reverse run is not exactly mirroring the previous forward run, but their curvatures can be different. This leads to a significant net displacement of the bacterium during movement.

**FIGURE 4:**
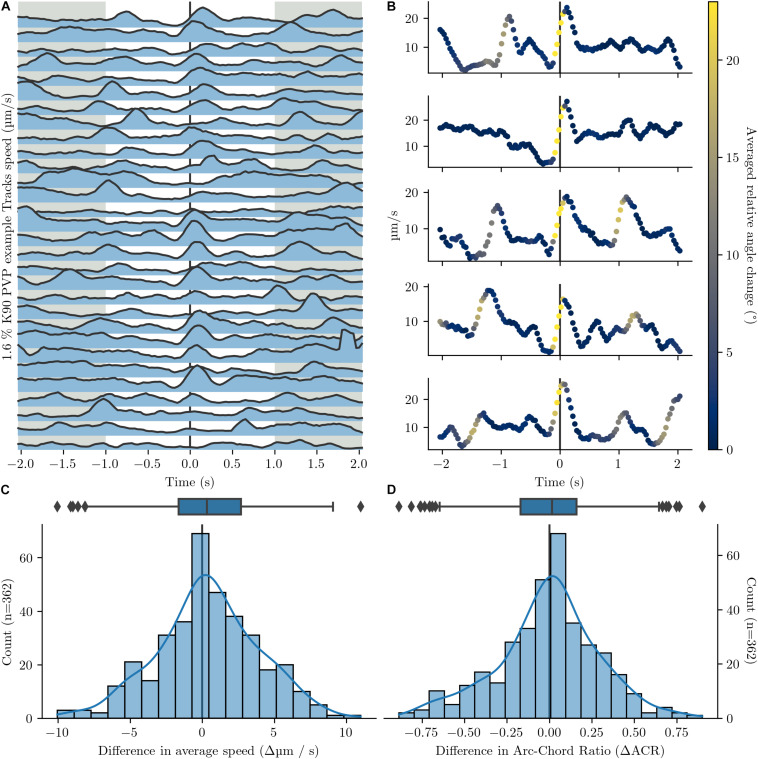
Comparison of speed and arc-chord ratio before and after a turnaround. Events with more than one calculated turnaround within a 4 s window were excluded. Turnarounds are set to the point with local greatest change in angle. Tracks are from Figure 1C. **(A)** Speed profile of 30 example tracks before and after a turnaround (black line, *T* = 0). **(B)** Five additional detailed speed graphs before and after turnaround (black line, *T* = 0). The colouration indicates the relative angle change between positions. The speed maximum at or up to 0.5 s after the turnaround ranges between 16.3 and 27.2 μm/s. **(C,D)** Histograms and corresponding box plots on the speed delta **(C)** and arc-chord ratio delta **(D)** before and after turnaround. The deltas are calculated between the time intervals [-2 s, -1 s] and [1 s, 2 s] (grey regions in **A**) around the turnaround.

A manual inspection of bacterial tracks revealed no obvious difference between a forward and a backward run phase. In addition, we performed a thorough quantitative comparison of two motility parameter, speed and arc-chored ratio, before and after a stop phase. We calculated the difference in average speed and in average arc-chored ratio before and after the stop for > 300 of these events and plotted the obtained difference values (Figure 4). If back and forth runs possess non-identical motility parameter, a histogram of difference values would show two peaks. In contrast, the histogram shows a typical Gaussian distribution, which is indicative for identical speed values and arc-chord ratios before and after a stop. This confirms that the two run phases between a stop are indistinguishable in their motility parameters.

In summary, this characterisation of the *C. difficile* swimming phenotype reveals a novel motility type for peritrichous bacteria.

## Discussion

### The Motility of *C. difficile* Is Dependent on Matrix Elasticity

We used single cell video microscopy in conjunction with a software-based quantification of motility parameters to characterise the motility profile of *C. difficile* under various conditions. The applied YSMR software allowed the unbiased and automated analysis of a large number of tracks per frame. It is well investigated that the motility of bacteria depends on the physical properties of the medium in which they move (Gao et al., 2014; Martinez et al., 2014; Bartlett et al., 2017; Taylor et al., 2019). When analysed in regular, water-based media without increased viscosity or matrix elasticity, *C. difficile* displayed a seemingly useless motility phenotype. Moving rapidly back and forth while achieving no notable displacement, the motility seemed to be highly energy intensive, yet ineffectual. It is unclear whether this motility phenotype has any relevance for *C. difficile* physiology and it is possible that it does not occur under natural environmental conditions.

Important physical characteristics shaping the environment include the viscosity, as well as the matrix elasticity of the medium (Gao et al., 2014; Martinez et al., 2014). Viscosity is generally accepted to be a major criterion for bacterial movement, as it plays an overwhelming role in the physics governing motility at the Reynolds number at which bacteria exist (Purcell, 1977). Increasing the medium viscosity by adding glycerol was therefore a logical first step to study motility. However, glycerol, which increases medium viscosity without affecting the matrix elasticity, did not strongly affect the swimming behaviour of *C. difficile* and effective motility could not be observed. In contrast, increase of matrix elasticity by PVP, a long-chained polymer at 360 MDa, or by mucins, both of which can form gel like structures, has a dramatic, concentration dependent effect on the swimming motility of *C. difficile*.

At PVP concentrations of 1.6%, a marked change in motility behaviour becomes visible with long, sustained run phases with varying curvature and intermittent turnarounds starting to appear that result in a net displacement of the bacterium. An optimum in terms of displacement/bacterial length ratio, speed, and arc-chord ratio is achieved at 3.2–3.6% (w/v) PVP. At 6.4% a decrease or complete cessation of movement can be observed, in which the strongly reduced number of motile cells perform very straight runs.

Our findings lead to the conclusion that the motility apparatus in *C. difficile*, across all clades, is adapted to an environment of large, high molarity molecules, which increase the matrix elasticity. Such molecules can be found in the natural environment of *C. difficile*, for example in the mucin-rich lower intestines. Association and interaction of *C. difficile* with the mucosal layer has been researched previously. Its ability to adhere to, and survive in the mucosal layer, as well as its demonstrated presence in the outer mucosal layer during infection, further indicates that the *C. difficile* motility apparatus is specialised for this environment (Tasteyre et al., 2001; Semenyuk et al., 2015; Soavelomandroso et al., 2017). It should be noted that Mucin types differ in their protein and glycosylation patterns. The bovine type I-S mucin used here is different from the human gastrointestinal mucin.

### *C. difficile* Displays a New Swimming Strategy

Depending on the species and the number and location of flagella across the cell body, bacteria use different swimming strategies. At least four different swimming types are described in detail in the literature, but none of them matches the observed *C. difficile* swimming strategy. The swimming pattern of the peritrichous model organism *E. coli* and its regulation are particularly well investigated. *E. coli* switches between a “run” mode and a “tumble” mode (Sarkar et al., 2010). During the run mode, the counter-clockwise rotating flagella form a bundle that pushes the cell forward, leading to a straight swimming phase. In the “tumble” mode the clockwise rotation of flagella leads to a breakup of the flagellar bundle, followed by a random change of the cell orientation. Bacteria with a single flagellum, such as *Vibrio alginolyticus* and *Rhodobacter sphaeroides*, possess different swimming patterns. *V. alginolyticus* displays a three-step pattern, in which a forward swimming phase is followed by a reverse swimming phase and a reorientation (“flick”) phase (Stocker, 2011; Xie et al., 2011). The flagellum of *R. sphaeroides* rotates in only one direction and the motility pattern follows a “stop and coil” pattern (Armitage and Macnab, 1987). Random reorientation occurs when the flagellar rotation stops. *Pseudomonas aeruginosa* performs a run-reverse-pause pattern, where reversals are marked with ∼180° angles. After pauses, the swimming direction does generally not change. Forward and backward travel directions do not differ and are achieved by clockwise or counter-clockwise turning of the single flagellum (Cai et al., 2016). Recently, a similar swimming strategy was identified in the singly flagellated proteobacterium *Caulobacter crescentus*, suggesting that the forward and backward motility pattern is more widely spread than previously expected (Grognot and Taute, 2021a). In fact, the “run and tumble” strategy of *E. coli* has not been identified for any polar flagellated bacterium (Grognot and Taute, 2021a). *Pseudomonas putida* produces a tuft of flagella on one cell pole. It can switch between a pushing mode, a pulling mode and a wrapping mode, with reorientation taking place during switches in modes (Hintsche et al., 2017).

The PVP-induced *C. difficile* motility pattern appears to be markedly different from those previously described. In particular, an obvious reorientation phase caused by a “tumble” or a “flick” appears to be absent. Instead, *C*. *difficile* stops in place at the end of a run phase and performs a ∼180° turnaround by changing the movement direction from forward to backward. Turnarounds therefore lead to a direct reversal in direction, but do not play an immediate role in the random reorientation process. While at a first glance this is similar to the polar monotrichous *Pseudomonas aeruginosa*, *C*. *difficile* has peritrichous flagella, which leads to the question how the reversal is achieved (Lawson et al., 2016; Anjuwon-Foster and Tamayo, 2017). At the beginning of a backward run *C*. *difficile* travel back on a very similar path as the previous forward run. However, the curvature can change during the course of the backward run, leading to a significant net displacement of the bacterium during movement.

Generally, flagella have an energetically favourable turning direction (Antani et al., 2021). In some species a difference in torque profile based on rotational direction, likely due to stator-rotor interactions, could be shown, which leads to different motility parameter for clockwise/counter-clockwise rotation (Yuan et al., 2010; Minamino et al., 2019). The energetically favourable turning direction can translate into a preferred direction of travel. In *C. difficile*, forward and backward runs are indistinguishable, suggesting that *C. difficile* has no preferred direction of travel. *C*. *difficile* is able to traverse bi-directional, which appears to be a novel mode of motility for peritrichous bacteria (Grognot and Taute, 2021b). These results suggest that matrix elasticity affects the *C. difficile* flagellar motor rotation state with a strong reduction of switching frequency in an environment of high matrix elasticity. Our findings lead to the conclusion that the motility apparatus in *C. difficile* is adapted to an environment of large, high molarity molecules with the ability for bi-directional motility.

## Data Availability Statement

The original contributions presented in the study are included in the article/Supplementary Material, further inquiries can be directed to the corresponding author/s.

## Author Contributions

JS, WB, and IO: conceptualisation, methodology, and validation. JS: software, formal analysis, data curation, and visualisation. JS and IO: investigation. UG, AZ, and WB: resources, supervision, and project administration. JS and WB: writing—original draft preparation. JS, UG, AZ, and WB: writing—review and editing. UG: funding acquisition. All authors contributed to the article and approved the submitted version.

## Conflict of Interest

The authors declare that the research was conducted in the absence of any commercial or financial relationships that could be construed as a potential conflict of interest.

## Supplementary Material

Supplementary Figure 1 Single cell motility dependency on PVP of *C. difficile* 630 Δ*erm*. The motility of *C. difficile* cells was tracked in the presence of six increasing K90 PVP concentrations (w/v) from 0.4 to 6.4% **(A–F)**. All observation durations were limited to 10 s for better comparability. The number of displayed tracks is stated in the title of each experiment. The starting position of each track is at the origin of the plot at position 0 μm/0 μm. The scale is identical for all six plots. Track colours denote the total travelled distance, with a colour bar on the right-hand side as a legend. A 5 μm scale bar is depicted in the lower left of subfigure **(A)**. The median cell lengths of *C. difficile* was determined to be between 4.81 and 5.28 μm (Supplementary Table 1).

Supplementary Figure 2 Direct comparison of motility characteristics and statistical distribution within experiments for different PVP concentrations. The motility of *C. difficile* was tracked in the presence of six increasing K90 polyvinylpyrrolidone (PVP) concentrations. The PVP concentration starts at 0.4% and increase at a doubling rate up to 6.4%, with an additional step of 3.6% PVP. All observations were limited to 10 s for better comparability. Average speed **(A)** and arc-chord ratio (**B**, denoting tortuosity of path) are plotted against the displacement/bacterial length ratio (showing the maximal distance in body lengths each bacterium has covered) for each tracked bacterium. The kernel density estimate is plotted independently for each experiment and variable, denoting the distribution for each characteristic.

Supplementary Figure 3 Single cell motility dependency on PVP of *C. difficile* 630 Δ*erm*. The motility of *C. difficile* cells was tracked in the presence of six increasing K90 PVP concentrations (w/v) from 0.4–6.4% **(A–F)**. All observation durations were limited to 10 s for better comparability. The number of displayed tracks is stated in the title of each experiment. The starting position of each track is at the origin of the plot at position 0 μm/0 μm. The scale is identical for all six plots. Track colours denote the total travelled distance, with a colour bar on the right-hand side as a legend. A 5 μm scale bar is depicted in the lower left of subfigure **(A)**. The median cell lengths of *C. difficile* was determined to be between 4.99 and 5.74 μm (Supplementary Table 1).

Supplementary Figure 4 Direct comparison of motility characteristics and statistical distribution within experiments for different PVP concentrations. The motility of *C. difficile* was tracked in the presence of six increasing K90 polyvinylpyrrolidone (PVP) concentrations. The PVP concentration starts at 0.4% and increase at a doubling rate up to 6.4%, with an additional step of 3.6% PVP. All observations were limited to 10 s for better comparability. Average speed **(A)** and arc-chord ratio (**B**, denoting tortuosity of path) are plotted against the displacement/bacterial length ratio (showing the maximal distance in body lengths each bacterium has covered) for each tracked bacterium. The kernel density estimate is plotted independently for each experiment and variable, denoting the distribution for each characteristic.

Supplementary Figure 5 Comparison of speed and arc-chord ratio before and after a turnaround. Events with more than one calculated turnaround within a 4 s window were excluded. Turnarounds are set to the point with local greatest change in angle. Tracks are from Figure 1C. **(A)** speed profile of 30 example tracks before and after a turnaround (black line, *T* = 0). **(B)** Five additional detailed speed graphs before and after turnaround (black line, *T* = 0). The colouration indicates the relative angle change between positions. The speed maximum at or up to 0.5 s after the turnaround ranges between 7.6 and 19.3 μm/s. **(C,D)** Histograms and corresponding box plots on the speed delta **(C)** and arc-chord ratio delta **(D)** before and after turnaround. The deltas are calculated between the time intervals [-2 s, -1 s] and [1 s, 2 s] (grey regions in **A**) around the turnaround.

Supplementary Figure 6 Comparison of speed and arc-chord ratio before and after a turnaround. Events with more than one calculated turnaround within a 4 s window were excluded. Turnarounds are set to the point with local greatest change in angle. Tracks are from Figure 1C. **(A)** speed profile of 30 example tracks before and after a turnaround (black line, *T* = 0). **(B)** Five additional detailed speed graphs before and after turnaround (black line, *T* = 0). The colouration indicates the relative angle change between positions. The speed maximum at or up to 0.5 s after the turnaround ranges between 2.5 and 20.0 μm/s. **(C,D)** Histograms and corresponding box plots on the speed delta **(C)** and arc-chord ratio delta **(D)** before and after turnaround. The deltas are calculated between the time intervals [-2 s, -1 s] and [1 s, 2 s] (grey regions in **A**) around the turnaround.

Supplementary Figure 7 Single cell motility dependency on mucin concentration of *C. difficile* 630 Δ*erm*. The motility of *C. difficile* cells was tracked in the presence of 5 increasing mucin concentrations. The mucin concentration starts at 2.5 mg/ml and increases in doubling steps up to 40 mg/ml **(A–E)**. The number of observations is stated in the title of each experiment. All observation durations were limited to 10 s for better comparability. The starting position of single *C. difficile* tracks are centred on the origin. The scale is identical for all six plots. Track colours denote the total travelled distance, with a colour bar on the right-hand side as a legend. A 5 μm scale bar is depicted in the lower left of subfigure **(A)**.

Supplementary Figure 8 Single cell motility dependency on mucin concentration of *C. difficile* 630 Δ*erm*. The motility of *C. difficile* cells was tracked in the presence of 5 increasing mucin concentrations. The mucin concentration starts at 2.5 mg/ml and increases in doubling steps up to 40 mg/ml **(A–E)**. The number of observations is stated in the title of each experiment. All observation durations were limited to 10 s for better comparability. The starting position of single *C. difficile* tracks are centred on the origin. The scale is identical for all six plots. Track colours denote the total travelled distance, with a colour bar on the right-hand side as a legend. A 5 μm scale bar is depicted in the lower left of subfigure **(A)**.

Supplementary Figure 9 Single cell motility dependency on mucin concentration of *C. difficile* 630 Δ*erm*. The motility of *C. difficile* cells was tracked in the presence of 5 increasing mucin concentrations. The mucin concentration starts at 2.5 mg/ml and increases in doubling steps up to 40 mg/ml **(A–E)**. The number of observations is stated in the title of each experiment. All observation durations were limited to 10 s for better comparability. The starting position of single *C. difficile* tracks are centred on the origin. The scale is identical for all six plots. Track colours denote the total travelled distance, with a colour bar on the right-hand side as a legend. A 5 μm scale bar is depicted in the lower left of subfigure **(A)**.

Supplementary Table 1 Various statistics on individual experiments. Values are given as: average (25 % quantile, median, 75 % quantile). N: number of observations.

Supplementary Movie 1 Motility of various *C. difficile* strains in BHIS without PVP. Video was taken with a 10x objective and converted to inverted grey-scale while filming. Shown are 630 Δ*erm* (clade 1), DSM 100002 (clade 1), R20291 (clade 2), DSM 102978 (clade 3), DSM 28670 (clade 4), and DSM 100005 (clade 4) in 100% BHIS.

Supplementary Movie 2 Motility in 10% BHIS/PBS with 3.6% PVP. The video of *C*. *difficile* 630 Δ*erm* was taken with a 64x objective.

Supplementary Movie 3 Motility in 10% BHIS/PBS with 2.5–40 mg/ml Mucin. Shown are excerpts of movies used in Supplementary Figure 7. The video of *C*. *difficile* 630 Δ*erm* was taken with a 10x objective and converted to inverted grey-scale while filming.

Supplementary Movie 4 Motility in 10% BHIS/PBS with 1.8% PVP and annotated tracks. The video of *C*. *difficile* 630 Δ*erm* was taken with a 10x objective and converted to inverted grey-scale while filming. Examples of tracks are depicted in Figure 3.

Supplementary Movie 5 Parts of Movies used in Figures 1, 2. The videos of *C*. *difficile* 630 Δ*erm* were taken with a 10x objective and converted to inverted grey-scale while filming. Shown are excerpts of movies of *C*. *difficile* 630 Δ*erm* in increasing PVP concentrations.

Supplementary Movie 6 Parts of Movies used in Supplementary Figures 1, 2. The videos of *C*. *difficile* 630 Δ*erm* were taken with a 10x objective and converted to inverted grey-scale while filming. Shown are excerpts of movies of *C*. *difficile* 630 Δ*erm* in increasing PVP concentrations.

Supplementary Movie 7 Parts of Movies used in Supplementary Figures 3, 4. The videos of *C*. *difficile* 630 Δ*erm* were taken with a 10x objective and converted to inverted grey-scale while filming. Shown are excerpts of movies of *C*. *difficile* 630 Δ*erm* in increasing PVP concentrations.

Supplementary Movie 8 Motility of various *C. difficile* strains in BHIS with PVP. The videos were taken with a 10x objective and converted to inverted grey-scale while filming. Shown are DSM 100002 (clade 1), R20291 (clade 2), DSM 102978 (clade 3), DSM 28670 (clade 4), and DSM 100005 (clade 4) with 2% (w/v) PVP.

Supplementary Text 1 Settings-file used for YSMR. “Supplementary_text 1_tracking.txt”.

Supplementary Text 2 Mucin/PVP handling and storage.
