# Supplementary material for: Clostridioides difficile Single Cell Swimming Strategy: A Novel Motility Pattern Regulated by Viscoelastic Properties of the Environment
Source: Front Microbiol. 2021 Jul 21;12:715220. doi: 10.3389/fmicb.2021.715220 (PMC8333305; doi:10.3389/fmicb.2021.715220)
Supplement: Supplementary Figure 1 — Single cell motility dependency on PVP of C. difficile 630 Δerm. The motility of C. difficile cells was tracked in the presence of six increasing K90 PVP concentrations (w/v) from 0.4 to 6.4% (A–F). All observation durations were limited to 10 s for better comparability. The number of displayed tracks is stated in the title of each experiment. The starting position of each track is at the origin of the plot at position 0 μm/0 μm. The scale is identical for all six plots. Track colours denote the total travelled distance, with a colour bar on the right-hand side as a legend. A 5 μm scale bar is depicted in the lower left of subfigure (A). The median cell lengths of C. difficile was determined to be between 4.81 and 5.28 μm (Supplementary Table 1). [file Presentation_1.pdf]

Supplementary Material

1 Supplemental Figures

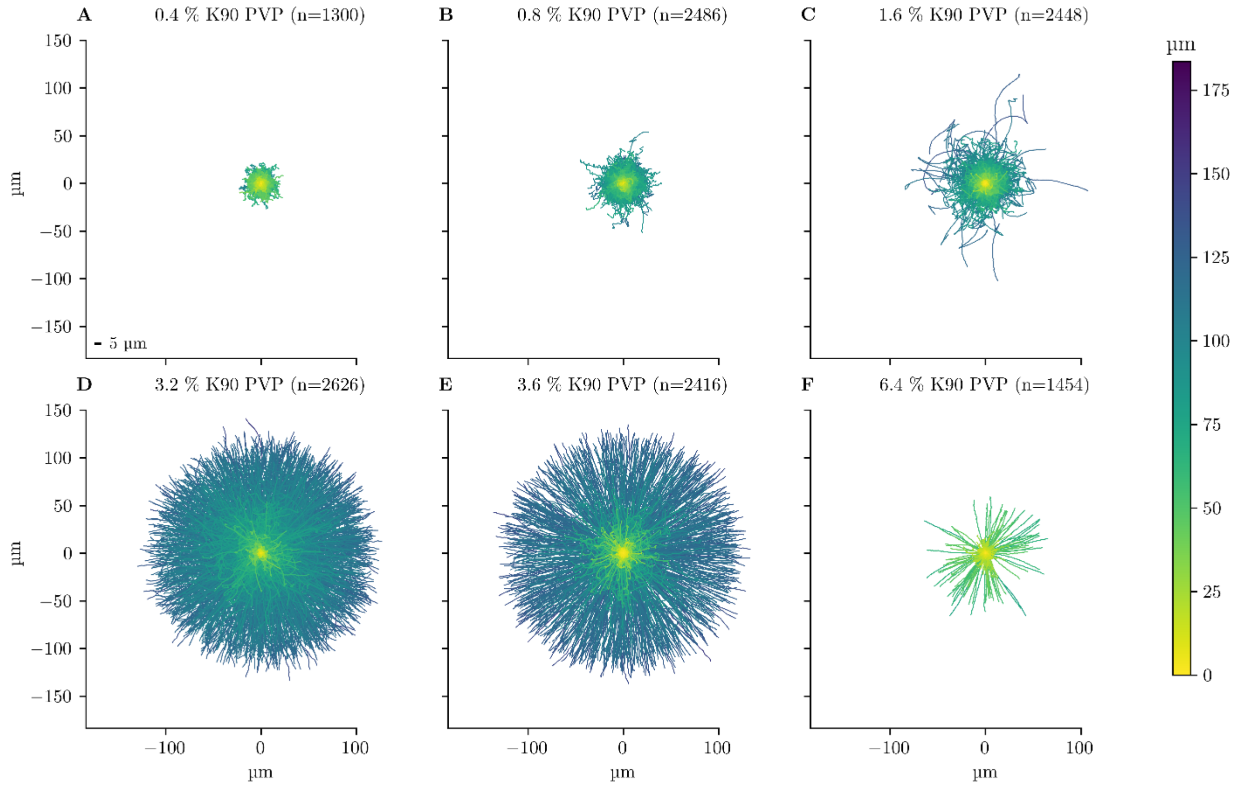

Supplemental Figure 1: Single cell motility dependency on PVP of *C. difficile* 630 Δ*erm*. The motility of *C. difficile* cells was tracked in the presence of six increasing K90 PVP concentrations (w/v) from 0.4%-6.4% (**A-F**). All observation durations were limited to 10 seconds for better comparability. The number of displayed tracks is stated in the title of each experiment. The starting position of each track is at the origin of the plot at position 0 µm/0µm. The scale is identical for all six plots. Track colours denote the total travelled distance, with a colour bar on the right-hand side as a legend. A 5 µm scale bar is depicted in the lower left of subfigure **A**. The median cell lengths of *C. difficile* was determined to be between 4.81 and 5.28 µm (**suppl. Table 1**).

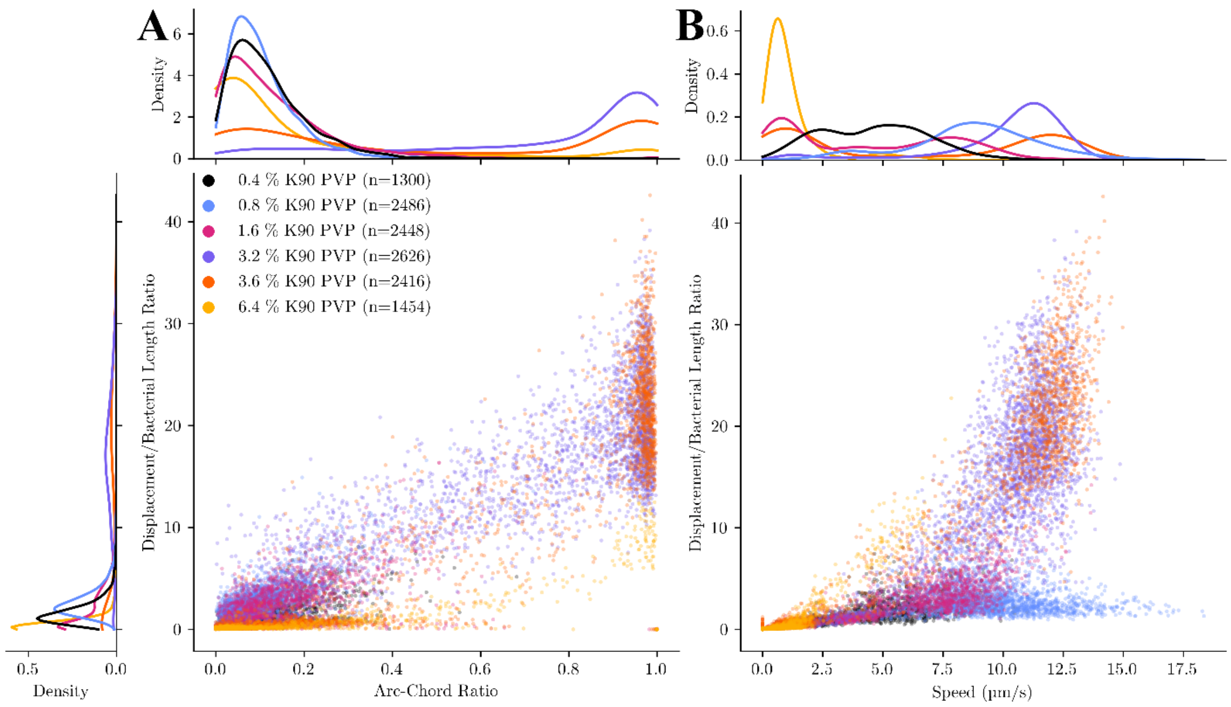

Supplemental Figure 2: Direct comparison of motility characteristics and statistical distribution within experiments for different PVP concentrations. The motility of *C. difficile* was tracked in the presence of six increasing K90 polyvinylpyrrolidone (PVP) concentrations. The PVP concentration starts at 0.4 % and increase at a doubling rate up to 6.4 %, with an additional step of 3.6 % PVP. All observations were limited to 10 seconds for better comparability. Average speed (**A**) and arc-chord ratio (**B**, denoting tortuosity of path) are plotted against the displacement/bacterial length ratio (showing the maximal distance in body lengths each bacterium has covered) for each tracked bacterium. The kernel density estimate is plotted independently for each experiment and variable, denoting the distribution for each characteristic.

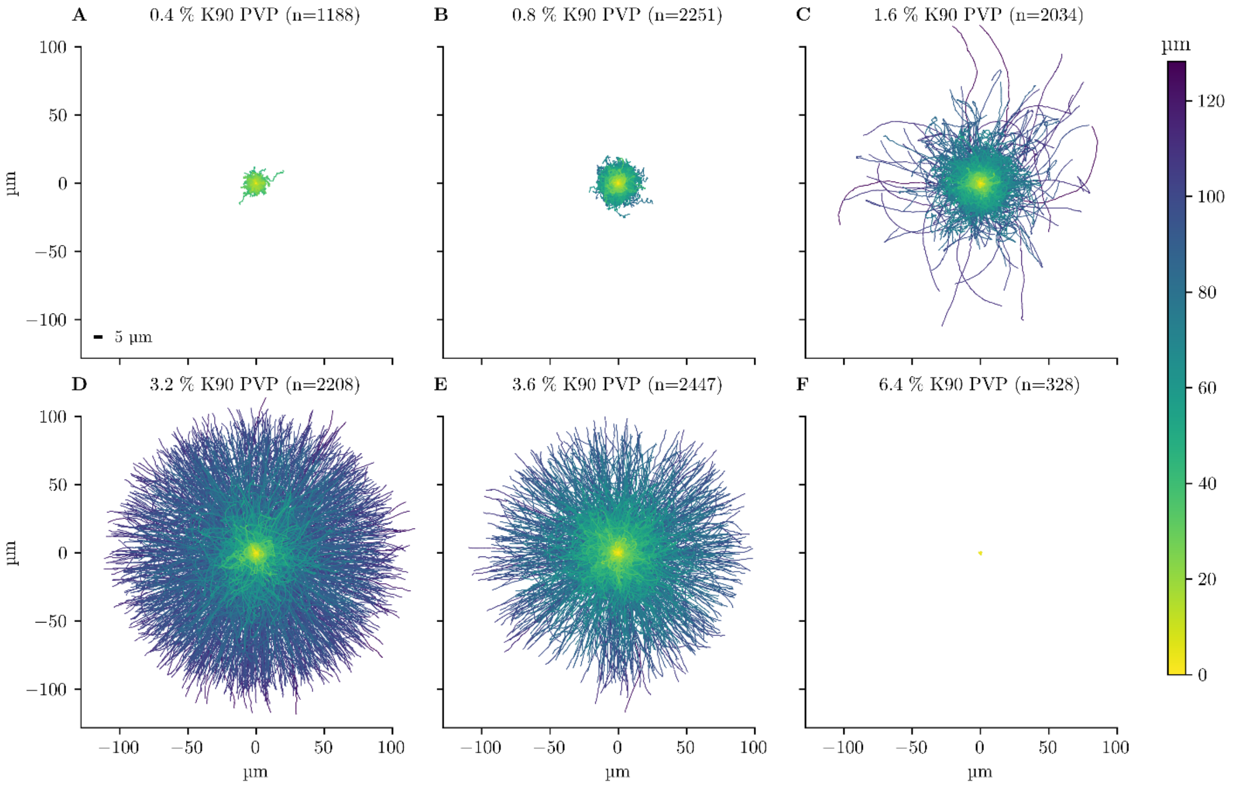

Supplemental Figure 3: Single cell motility dependency on PVP of *C. difficile* 630 Δ*erm*. The motility of *C. difficile* cells was tracked in the presence of six increasing K90 PVP concentrations (w/v) from 0.4%-6.4% (**A-F**). All observation durations were limited to 10 seconds for better comparability. The number of displayed tracks is stated in the title of each experiment. The starting position of each track is at the origin of the plot at position 0 µm/0µm. The scale is identical for all six plots. Track colours denote the total travelled distance, with a colour bar on the right-hand side as a legend. A 5 µm scale bar is depicted in the lower left of subfigure **A**. The median cell lengths of *C. difficile* was determined to be between 4.99 and 5.74 µm (**suppl. Table 1**).

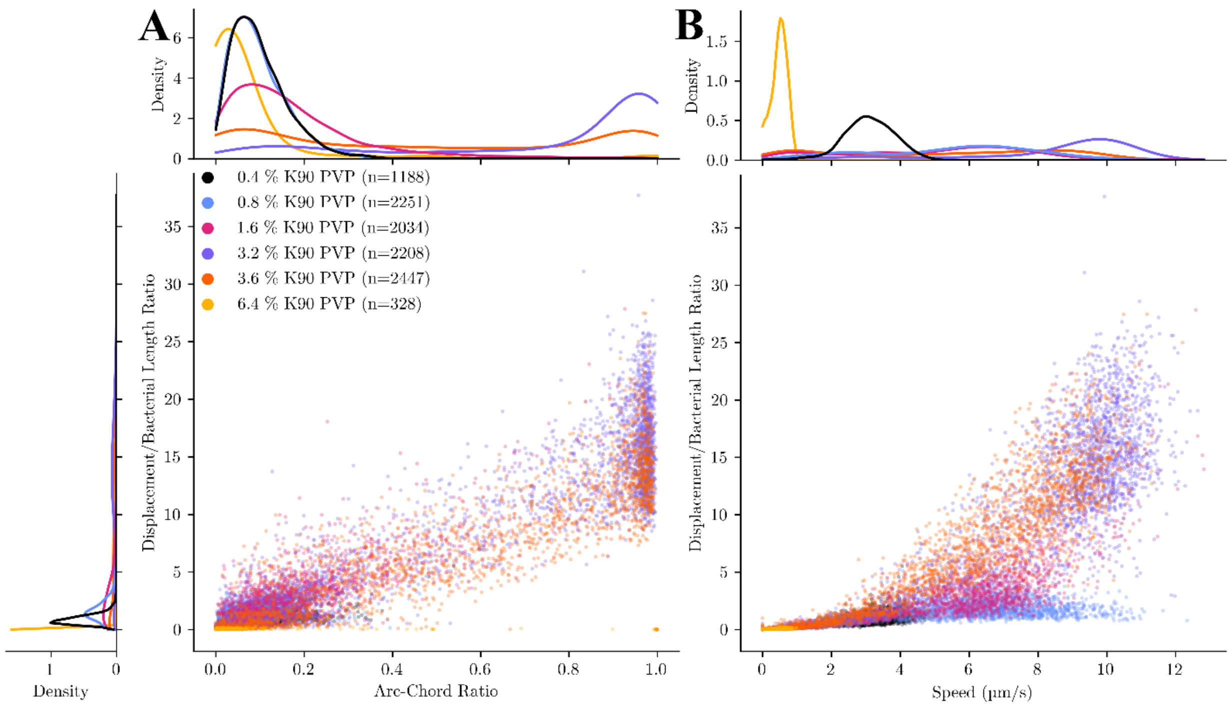

Supplemental Figure 4: Direct comparison of motility characteristics and statistical distribution within experiments for different PVP concentrations. The motility of *C. difficile* was tracked in the presence of six increasing K90 polyvinylpyrrolidone (PVP) concentrations. The PVP concentration starts at 0.4 % and increase at a doubling rate up to 6.4 %, with an additional step of 3.6 % PVP. All observations were limited to 10 seconds for better comparability. Average speed (**A**) and arc-chord ratio (**B**, denoting tortuosity of path) are plotted against the displacement/bacterial length ratio (showing the maximal distance in body lengths each bacterium has covered) for each tracked bacterium. The kernel density estimate is plotted independently for each experiment and variable, denoting the distribution for each characteristic.

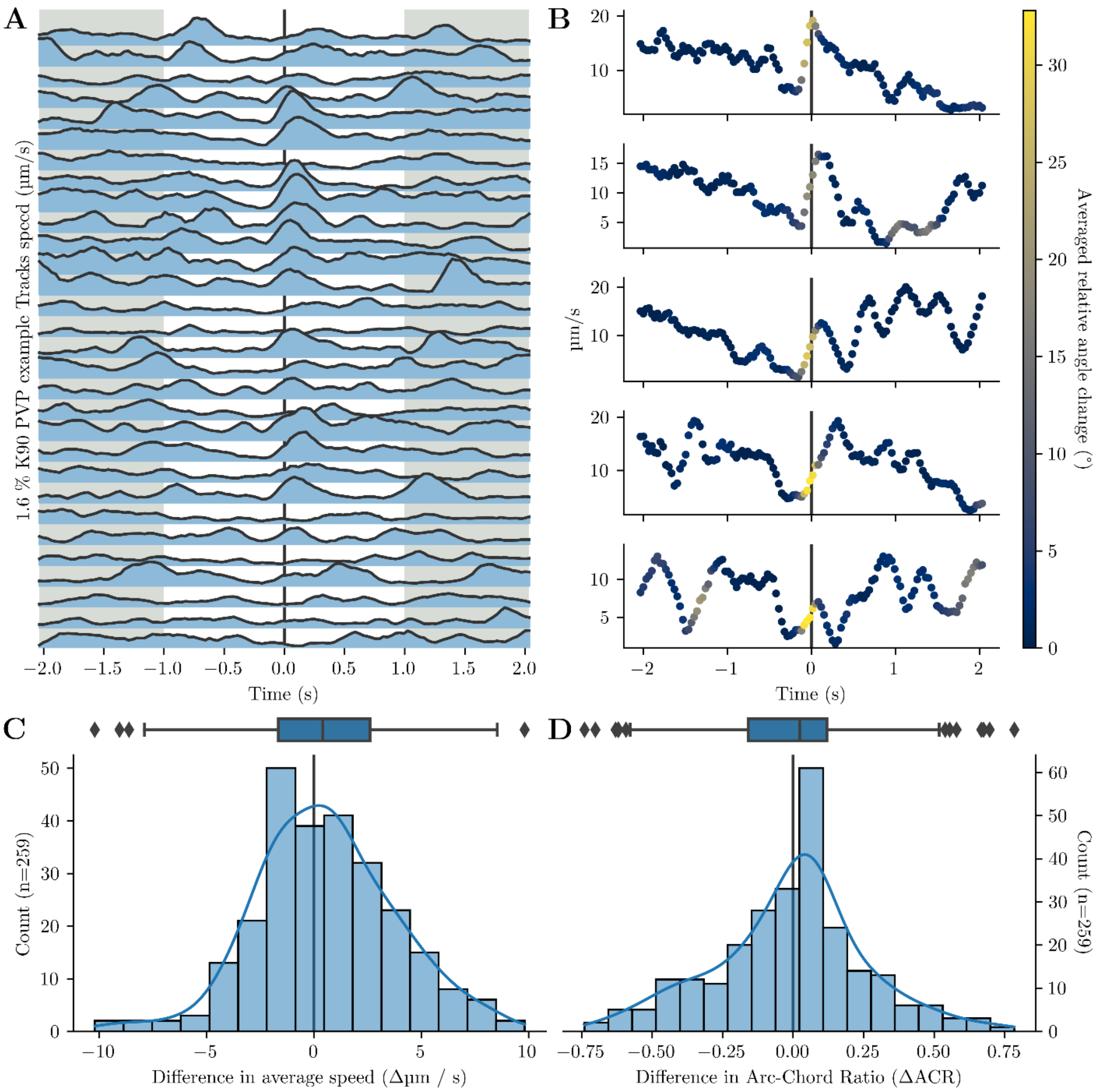

Supplemental Figure 5: Comparison of speed and arc-chord ratio before and after a turnaround. Events with more than one calculated turnaround within a 4 s window were excluded. Turnarounds are set to the point with local greatest change in angle. Tracks are from **Figure 1 C**. **A**: speed profile of 30 example tracks before and after a turnaround (black line, T = 0). **B**: Five additional detailed speed graphs before and after turnaround (black line, T = 0). The coloration indicates the relative angle change between positions. The speed maximum at or up to 0.5 s after the turnaround ranges between 7.6 and 19.3 µm/s. **C**, **D**: Histograms and corresponding box plots on the speed delta (**C**) and arc-chord ratio delta (**D**) before and after turnaround. The deltas are calculated between the time intervals [-2 s, -1 s] and [1 s, 2 s] (grey regions in **A**) around the turnaround.

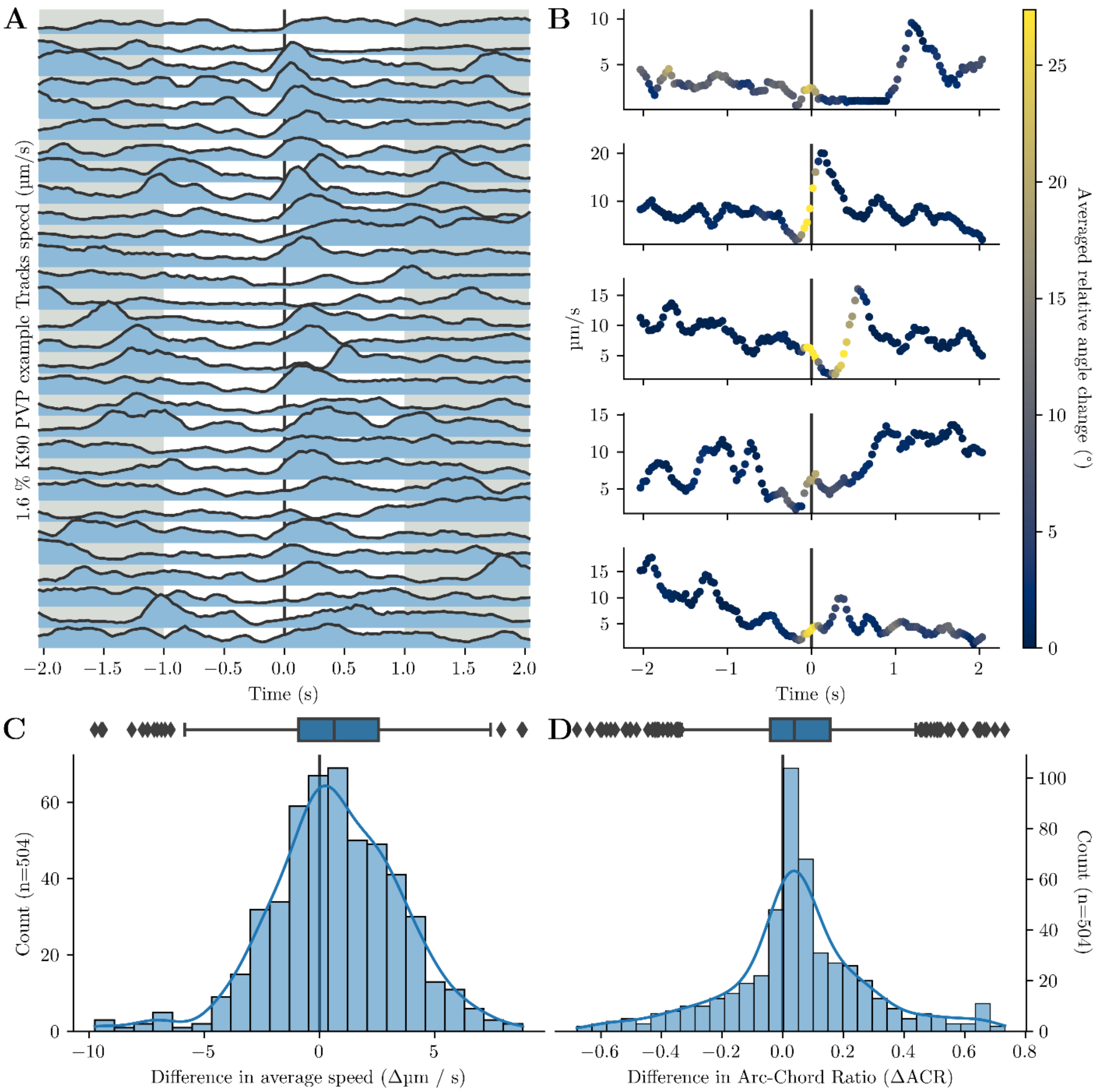

Supplemental Figure 6: Comparison of speed and arc-chord ratio before and after a turnaround. Events with more than one calculated turnaround within a 4 s window were excluded. Turnarounds are set to the point with local greatest change in angle. Tracks are from **Figure 1 C**. **A**: speed profile of 30 example tracks before and after a turnaround (black line, T = 0). **B**: Five additional detailed speed graphs before and after turnaround (black line, T = 0). The coloration indicates the relative angle change between positions. The speed maximum at or up to 0.5 s after the turnaround ranges between 2.5 and 20.0 µm/s. **C**, **D**: Histograms and corresponding box plots on the speed delta (**C**) and arc-chord ratio delta (**D**) before and after turnaround. The deltas are calculated between the time intervals [-2 s, -1 s] and [1 s, 2 s] (grey regions in **A**) around the turnaround.

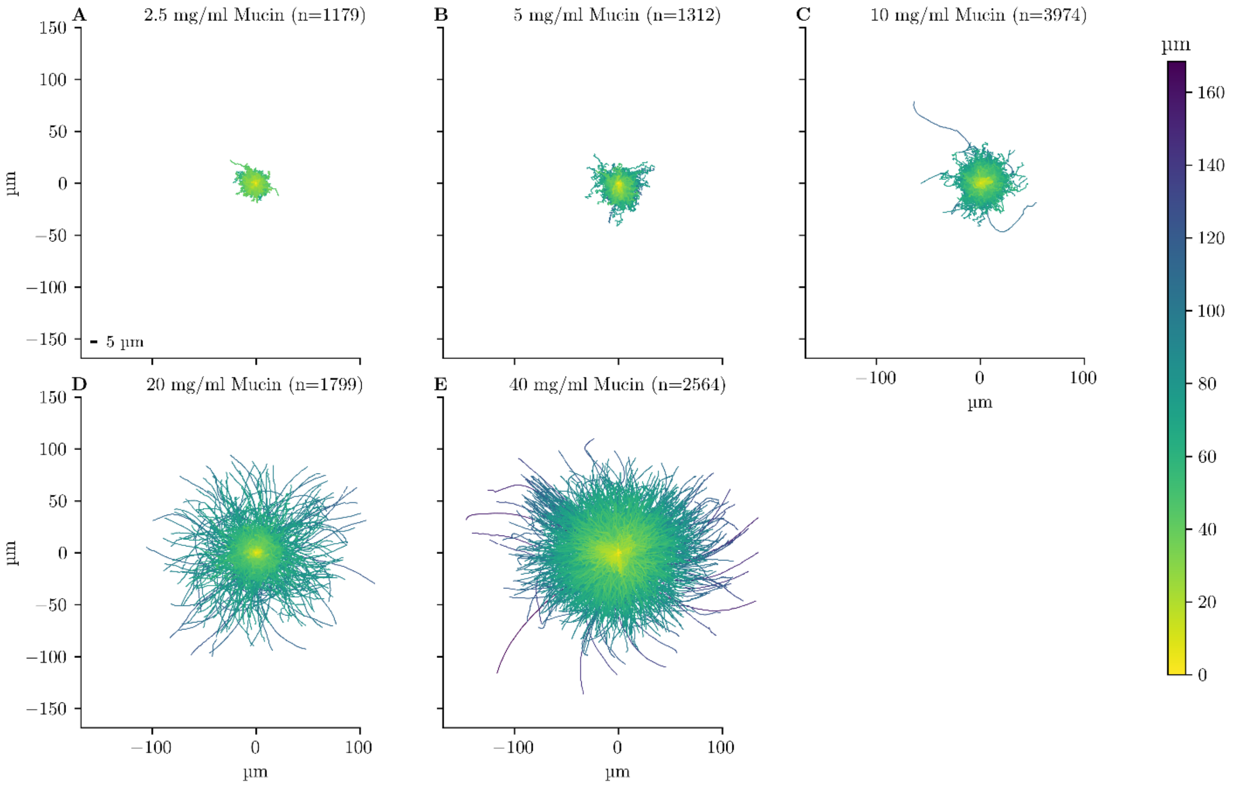

Supplemental Figure 7: Single cell motility dependency on mucin concentration of *C. difficile* 630 Δ*erm*. The motility of *C. difficile* cells was tracked in the presence of 5 increasing mucin concentrations. The mucin concentration starts at 2.5 mg/ml and increases in doubling steps up to 40 mg/ml (**A** – **E**). The number of observations is stated in the title of each experiment. All observation durations were limited to 10 seconds for better comparability. The starting position of single *C. difficile* tracks are centred on the origin. The scale is identical for all six plots. Track colours denote the total travelled distance, with a colour bar on the right-hand side as a legend. A 5 µm scale bar is depicted in the lower left of subfigure **A**.

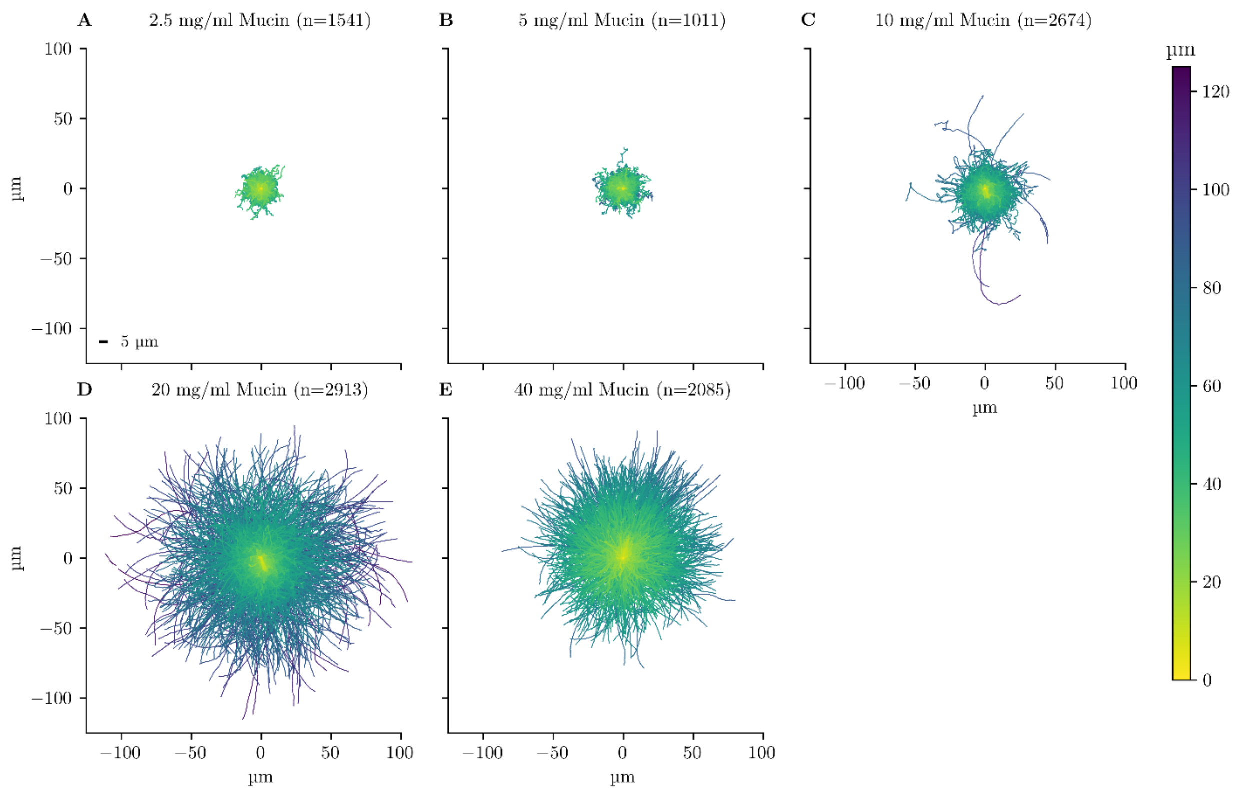

Supplemental Figure 8: Single cell motility dependency on mucin concentration of *C. difficile* 630 Δ*erm*. The motility of *C. difficile* cells was tracked in the presence of 5 increasing mucin concentrations. The mucin concentration starts at 2.5 mg/ml and increases in doubling steps up to 40 mg/ml (**A** – **E**). The number of observations is stated in the title of each experiment. All observation durations were limited to 10 seconds for better comparability. The starting position of single *C. difficile* tracks are centred on the origin. The scale is identical for all six plots. Track colours denote the total travelled distance, with a colour bar on the right-hand side as a legend. A 5 µm scale bar is depicted in the lower left of subfigure **A**.

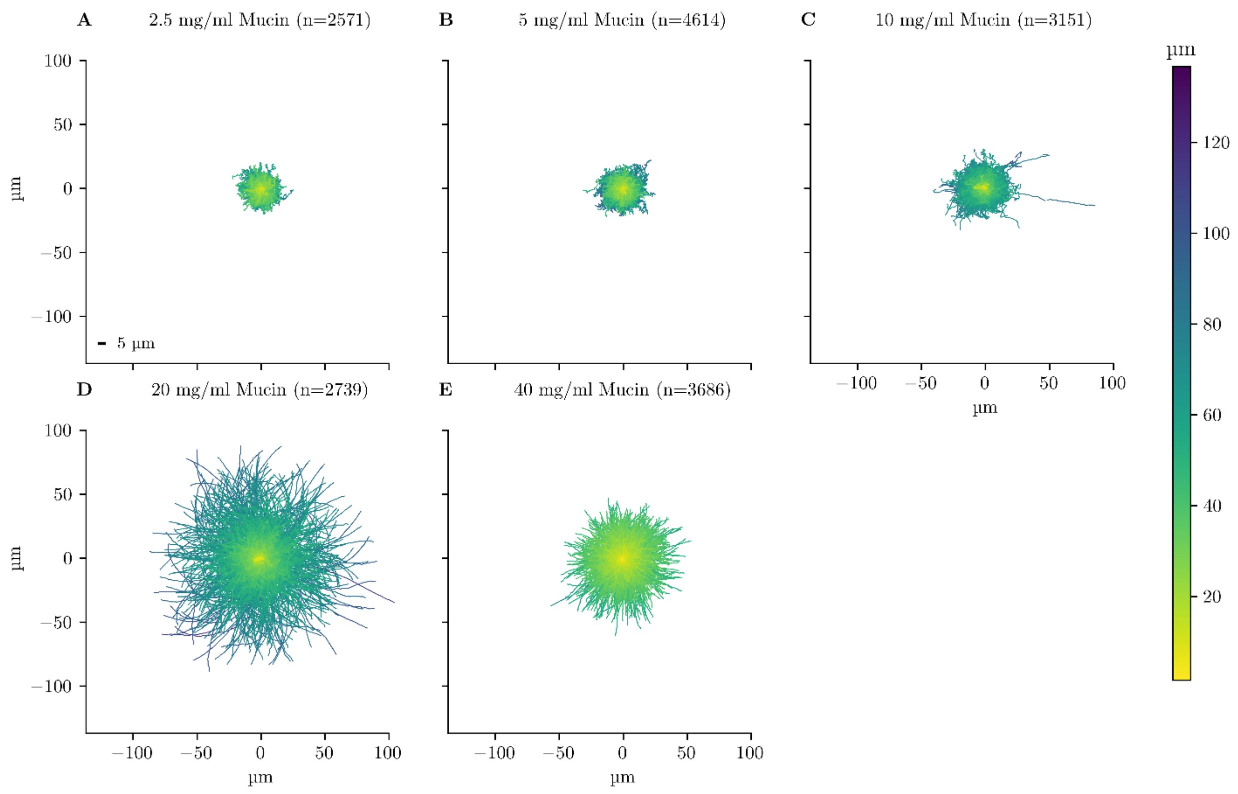

Supplemental Figure 9: Single cell motility dependency on mucin concentration of *C. difficile* 630 Δ*erm*. The motility of *C. difficile* cells was tracked in the presence of 5 increasing mucin concentrations. The mucin concentration starts at 2.5 mg/ml and increases in doubling steps up to 40 mg/ml (**A** – **E**). The number of observations is stated in the title of each experiment. All observation durations were limited to 10 seconds for better comparability. The starting position of single *C. difficile* tracks are centred on the origin. The scale is identical for all six plots. Track colours denote the total travelled distance, with a colour bar on the right-hand side as a legend. A 5 µm scale bar is depicted in the lower left of subfigure **A**.

2 Supplemental Tables

	Condition	Displacement (µm)	Displacement/ Bacterial Length Ratio	Distance (µm)	Speed (µm/s)	Arc-Chord Ratio	Bacteria Length
**Figure 1 & 2**	0.4 % K90 PVP (n=1271)	7.45 (4.76, 6.93, 9.62)	1.69 (0.93, 1.40, 2.19)	45.04 (37.66, 46.69, 56.16)	4.50 (3.77, 4.67, 5.62)	0.13 (0.06, 0.11, 0.17)	4.75 (3.78, 4.55, 5.59)
	0.8 % K90 PVP (n=1369)	11.03 (7.55, 10.32, 13.53)	2.68 (1.56, 2.35, 3.31)	81.36 (55.53, 74.84, 109.99)	8.14 (5.55, 7.48, 11.00)	0.11 (0.05, 0.09, 0.14)	4.51 (3.57, 4.30, 5.31)
	1.6 % K90 PVP (n=4326)	19.47 (12.48, 16.70, 23.15)	4.52 (2.68, 3.77, 5.40)	89.24 (80.70, 92.67, 104.19)	8.92 (8.07, 9.27, 10.42)	0.14 (0.07, 0.12, 0.18)	4.54 (3.61, 4.33, 5.33)
	3.2 % K90 PVP (n=3986)	35.79 (1.88, 5.08, 66.64)	7.39 (0.39, 1.15, 13.42)	55.23 (9.84, 21.24, 109.59)	5.52 (0.98, 2.12, 10.96)	0.34 (0.07, 0.22, 0.59)	4.81 (4.02, 4.65, 5.45)
	3.6 % K90 PVP (n=2580)	97.02 (73.14, 111.25, 125.75)	21.40 (15.99, 21.67, 27.86)	118.91 (113.57, 126.28, 135.70)	11.89 (11.36, 12.63, 13.57)	0.74 (0.57, 0.90, 0.96)	4.68 (3.82, 4.46, 5.40)
	6.4 % K90 PVP (n=2221)	82.64 (79.76, 87.83, 94.28)	16.52 (13.59, 16.80, 20.25)	85.92 (82.87, 90.50, 96.78)	8.59 (8.29, 9.05, 9.68)	0.91 (0.96, 0.97, 0.98)	5.24 (4.25, 5.07, 6.05)
**Suppl. Figure 1 & 2**	0.4 % K90 PVP (n=1300)	7.43 (3.93, 6.63, 9.98)	1.68 (0.86, 1.38, 2.14)	46.09 (27.35, 46.96, 62.08)	4.61 (2.74, 4.70, 6.21)	0.12 (0.05, 0.10, 0.16)	4.70 (3.74, 4.50, 5.44)
	0.8 % K90 PVP (n=2486)	13.17 (9.30, 12.23, 15.87)	2.86 (1.75, 2.41, 3.44)	87.63 (73.90, 88.77, 104.30)	8.76 (7.39, 8.88, 10.43)	0.11 (0.05, 0.09, 0.14)	4.98 (3.95, 4.81, 5.88)
	1.6 % K90 PVP (n=2448)	11.04 (1.19, 6.22, 16.92)	2.11 (0.23, 1.26, 3.24)	43.17 (8.76, 38.20, 75.72)	4.32 (0.88, 3.82, 7.57)	0.13 (0.04, 0.10, 0.18)	5.26 (4.39, 5.12, 6.04)
	3.2 % K90 PVP (n=2626)	83.16 (60.04, 95.83, 109.58)	16.06 (11.64, 16.57, 20.88)	101.56 (96.12, 109.04, 117.54)	10.16 (9.61, 10.90, 11.75)	0.73 (0.56, 0.90, 0.97)	5.33 (4.40, 5.18, 6.08)
	3.6 % K90 PVP (n=2416)	53.98 (2.23, 31.38, 113.42)	10.44 (0.44, 6.29, 20.25)	63.40 (9.79, 58.68, 117.85)	6.34 (0.98, 5.87, 11.79)	0.52 (0.10, 0.47, 0.97)	5.19 (4.36, 5.06, 5.92)
	6.4 % K90 PVP (n=1454)	5.17 (0.53, 0.81, 1.69)	0.94 (0.10, 0.16, 0.34)	11.46 (4.55, 7.70, 12.56)	1.15 (0.45, 0.77, 1.26)	0.18 (0.02, 0.06, 0.19)	5.37 (4.45, 5.28, 6.23)
**Suppl. Figure 3 & 4**	0.4 % K90 PVP (n=1188)	4.29 (3.08, 3.98, 5.19)	0.90 (0.55, 0.80, 1.12)	30.95 (26.42, 30.99, 36.01)	3.10 (2.64, 3.10, 3.60)	0.10 (0.05, 0.09, 0.13)	5.23 (4.03, 5.03, 6.24)
	0.8 % K90 PVP (n=2251)	8.09 (5.32, 8.02, 10.47)	1.65 (0.99, 1.48, 2.15)	54.57 (35.16, 58.36, 72.10)	5.46 (3.52, 5.84, 7.21)	0.10 (0.05, 0.08, 0.13)	5.22 (4.08, 4.99, 6.17)
	1.6 % K90 PVP (n=2034)	16.11 (5.11, 12.43, 20.43)	2.96 (1.07, 2.24, 3.88)	50.68 (29.89, 55.70, 70.42)	5.07 (2.99, 5.57, 7.04)	0.18 (0.07, 0.13, 0.23)	5.48 (4.33, 5.32, 6.38)
	3.2 % K90 PVP (n=2208)	70.04 (45.71, 84.85, 96.27)	12.13 (7.98, 12.85, 16.59)	82.54 (73.51, 93.01, 101.32)	8.25 (7.35, 9.30, 10.13)	0.74 (0.53, 0.92, 0.97)	5.94 (4.85, 5.74, 6.93)
	3.6 % K90 PVP (n=2447)	38.08 (4.05, 31.12, 71.14)	6.56 (0.79, 5.60, 11.18)	52.58 (20.84, 56.81, 81.39)	5.26 (2.08, 5.68, 8.14)	0.48 (0.11, 0.44, 0.90)	5.65 (4.58, 5.45, 6.60)
	6.4 % K90 PVP (n=328)	0.39 (0.29, 0.38, 0.46)	0.08 (0.05, 0.07, 0.10)	4.85 (3.57, 5.15, 6.50)	0.48 (0.36, 0.51, 0.65)	0.07 (0.01, 0.03, 0.05)	5.37 (4.30, 5.18, 6.29)

Supplemental Table 1: Various statistics on individual experiments. Values are given as: average (25 % quantile, median, 75 % quantile). N: number of observations.

3 Supplemental Videos:

Movie S1: Motility of various *C. difficile* strains in BHIS without PVP. Video was taken with a 10x objective. Shown are 630 Δ*erm* (clade 1), DSM 100002 (clade 1), R20291 (clade 2), DSM 102978 (clade 3), DSM 28670 (clade 4), and DSM 100005 (clade 4) in 100 % BHI.

Movie S2: Motility in 10 % BHIS/PBS with 3.6 % PVP. The video of *C. difficile* 630 Δ*erm* was taken with a 64x objective.

Movie S3: Motility in 10 % BHIS/PBS with 40 mg/ml Mucin. The video of *C. difficile* 630 Δ*erm* was taken with a 10x objective.

Movie S4: Motility in 10 % BHIS/PBS with 1.8 % PVP and annotated tracks. The video of *C. difficile* 630 Δ*erm* was taken with a 10x objective. Examples of tracks are depicted in **Fig. 3**.

Movie S5: Parts of Movies used in Figure 1 & 2. Shown are excerpts of movies of *C. difficile* 630 Δ*erm* in increasing PVP concentrations.

Movie S6: Parts of Movies used in supplemental Figure 1 & 2. Shown are excerpts of movies of *C. difficile* 630 Δ*erm* in increasing PVP concentrations.

Movie S7: Parts of Movies used in supplemental Figure 3 & 4. Shown are excerpts of movies of *C. difficile* 630 Δ*erm* in increasing PVP concentrations.

Movie S8: Motility of various *C. difficile* strains in BHIS with PVP. Video was taken with a 10x objective. Shown are DSM 100002 (clade 1), R20291 (clade 2), DSM 102978 (clade 3), DSM 28670 (clade 4), and DSM 100005 (clade 4) with 2 % (w/v) PVP.
