# Supplementary material for: Clostridioides difficile Single Cell Swimming Strategy: A Novel Motility Pattern Regulated by Viscoelastic Properties of the Environment
Source: Front Microbiol. 2021 Jul 21;12:715220. doi: 10.3389/fmicb.2021.715220 (PMC8333305; doi:10.3389/fmicb.2021.715220)
Supplement: Supplementary file 12 [file Data_Sheet_2.docx]

**K 90 polyvinylpyrrolidone (MW 360,000 g/mol, Carl Roth, Karlsruhe, Germany, order nr. CP15.1) Stock solution:**

In a 15 ml tube, for a 10 % stock solution, prepare 0.5 g PVP and fill up to 5 ml with PBS (PBS, Merck, Darmstadt, Germany, order nr. D5652).

PVP dissolves best when left on a tilt table or turntable at room temperature for several hours or overnight. PVP dissolves fully, if white precipitate is still present, increase the incubation time.

If a large bit of PVP powder is stuck in the tip of the 15 ml tube, dragging the tip vigorously over a 1.5 ml reaction tube stand or similarly spaced object repeatedly can loosen the powder and allow for better dissolution.

The stock solution is very viscous, but liquid enough for pipetting with a 200 µl tip when performed slowly enough.

**Type I-S mucin (Bovine Submaxillary glands, Merck, Darmstadt, Germany, order nr. M3895-100MG) Stock solution:**

Similarly, for Mucin, a 100 mg/ml stock solution is prepared:

Solve 0.5 g in 4 ml PBS and let dissolve on a tilt or turntable for several hours.

The pH should be checked and if necessary adjusted to 7.

Now adjust volume to 5 ml with PBS.

Sterile filtration is not advised, as this will nearly fully remove the mucin from the solution.

The stock solution should be stored at 4 °C and used within a few days of preparation, as otherwise precipitate starts to form.

Some debris is to be expected during microscopy. We have found that sonification or centrifugation is not helpful, or at worst decreases the mucin-concentration without affecting the debris.
